# Prone positioning as an emerging tool in the care provided to patients infected with COVID-19: a scoping review*

**DOI:** 10.1590/1518-8345.4732.3397

**Published:** 2021-01-08

**Authors:** Marília Souto de Araújo, Marina Marisa Palhano dos Santos, Carlos Jordão de Assis Silva, Rejane Maria Paiva de Menezes, Alexsandra Rodrigues Feijão, Soraya Maria de Medeiros

**Affiliations:** 1 Universidade Federal do Rio Grande do Norte, Natal, RN, Brazil.

**Keywords:** Coronavirus Infections, Respiratory Tract Infections, Adult Respiratory Distress Syndrome, Prone Position, Nursing, Critical Care, Infecções por Coronavírus, Infecções Respiratórias, Síndrome do Desconforto Respiratório do Adulto, Decúbito Ventral, Enfermagem, Cuidados Críticos, Infecciones por Coronavirus, Infecciones del Sistema Respiratorio, Síndrome de Dificultad Respiratoria del Adulto, Posición Prona, Enfermería, Cuidados Críticos

## Abstract

**Objective:**

to describe scientific evidence regarding the use of prone positioning in the care provided to patients with acute respiratory failure caused by COVID-19.

**Method:**

this is a scoping review. PRISMA Extension for Scoping Reviews was used to support the writing of this study. The search was conducted in seven databases and resulted in 2,441 studies, 12 of which compose the sample. Descriptive statistics, such as relative and absolute frequencies, was used to analyze data.

**Results:**

prone positioning was mainly adopted in Intensive Care Units, lasted from a minimum of 12 up to 16 hours, and its prescription was based on specific criteria, such as PaO_2_/FiO_2_ ratio, oxygen saturation, and respiratory rate. The most prevalent complications were: accidental extubation, pressure ulcer, and facial edema. Decreased hypoxemia and mortality rates were the main outcomes reported.

**Conclusion:**

positive outcomes outweighed complications. Various cycles of prone positioning are needed, which may cause potential work overload for the health staff. Therefore, an appropriate number of trained workers is necessary, in addition to specific institutional protocols to ensure patient safety in this context.

## Abstract (pt)

**Objetivo:**

descrever as evidências científicas acerca da utilização da posição prona na assistência ao paciente com insuficiência respiratória aguda provocada por COVID-19.

**Método:**

trata-se de uma *scoping review.* O instrumento PRISMA *Extension for Scoping Reviews* foi utilizado para a redação do estudo. As buscas foram realizadas em sete bases de dados, resultando em 2.441 estudos dos quais 12 compõem a amostra. Uma análise descritiva dos dados foi realizada empregando frequências relativas e absolutas.

**Resultados:**

a utilização da posição prona ocorreu principalmente em Unidades de Terapia Intensiva, com duração mínima de 12 a 16 horas, e teve como fundamentos de indicação critérios específicos, tais como a relação PaO_2_/FiO_2_, a saturação de oxigênio e a frequência respiratória. As complicações mais prevalentes da sua utilização foram: extubação acidental, lesão por pressão e edema facial. Identificou-se a redução da hipoxemia e da mortalidade como principais desfechos evidenciados na amostra.

**Conclusão:**

os desfechos positivos sobressaíram-se face às complicações. São necessários vários ciclos de pronação do paciente, fator causador de possível sobrecarga de trabalho da equipe de saúde. Portanto, são importantes um adequado dimensionamento dos profissionais, uma equipe treinada e protocolos institucionais específicos a fim de se garantir a segurança do paciente nesse contexto.

## Abstract (es)

**Objetivo:**

describir las evidencias científicas acerca de la utilización de la posición prona en la atención al paciente con insuficiencia respiratoria aguda provocada por COVID-19.

**Método:**

se trata de una revisión de escopo*.* El instrumento PRISMA *Extension for Scoping Reviews* fue utilizado para la redacción del estudio. Las búsquedas fueron realizadas en siete bases de datos, resultando en 2.441 estudios de los cuales 12 integran la muestra. Un análisis descriptivo de los datos fue desarrollado empleando frecuencias relativas y absolutas.

**Resultados:**

la utilización de la posición prona ocurrió principalmente en Unidades de Terapia Intensiva, con duración mínima de 12 a 16 horas, y tuvo como fundamentos de indicación criterios específicos, tales como la relación PaO_2_/FiO_2_, la saturación de oxígeno y la frecuencia respiratoria. Las complicaciones más frecuentes de su uso fueron: desintubación accidental, lesión por presión y edema facial. Se identificó la reducción de la hipoxemia y de la mortalidad como principales resultados evidenciados en la muestra.

**Conclusión:**

los resultados positivos se destacaran ante las complicaciones. Son necesarios varios ciclos de pronación del paciente, factor causante de una posible sobrecarga de trabajo del equipo de salud. Por lo tanto, son importantes un adecuado dimensionamiento de los profesionales, un equipo capacitado y protocolos institucionales específicos a fin de garantizar la seguridad del paciente en ese contexto.

## Introduction

During December 2019, the city of Wuhan, China witnessed an outbreak of pneumonia of unknown cause. In January 2020, Chinese scientists isolated the causative agent, the novel Coronavirus (SARS-CoV-2). In February of the same year, the World Health Organization (WHO) called this pathology COVID-19^(^ 1 ^)^.

The disease spread rapidly and became a concern due to the large number of individuals who were contaminated and died worldwide. A total of 19,266,406 cases and 718,530 deaths caused by COVID-19 were confirmed up to August 7^th^, 2020^(^ 2 ^)^. In Brazil, 2,967,064 cases and 99,702 deaths had been confirmed until the same date^(^ 2 ^)^.

COVID-19 is characterized by a broad clinical spectrum, encompassing asymptomatic infection, mild disease of the upper respiratory tract, and severe viral pneumonia with respiratory failure, multiple organ failure, and even death^(^ 3 ^)^. The most common symptoms at the onset of COVID-19 are fever, cough, and fatigue, while other symptoms include dyspnea, headache, hemoptysis, anosmia, dysgeusia, and diarrhea. In its most severe manifestation, clinical characteristics indicate the development of Acute Respiratory Discomfort Syndrome (ARDS), acute cardiac injury, and thrombotic phenomena^(^ 4 ^)^. One study^(^ 5 ^)^ confirms that the most common symptoms at the onset of the disease include fever (98%) and cough (76%); dyspnea was found in 55% of the patients. Complications inherent to the most severe form of the disease affected 29% of the patients, who developed ARDS, requiring critical care.

According to data of the Chinese Center for Disease Control and Prevention, which included 44,500 people with a confirmed diagnosis of the SARS-CoV-2 infection, 14% of the cases were affected by the most severe form of the disease, while those with a critical condition, presenting respiratory failure and consequently requiring mechanical ventilation, totaled 5%^(^ 6 ^)^.

Currently, the early intubation of patients with COVID-19 is recommended, especially among those with severe hypoxemia, characterized by a PaO_2_/FiO_2_ ratio <200 mmHg, according to the Berlin criteria for ARDS^(^ 7 ^)^. The literature recommends Prone Positioning (PP) associated with mechanical ventilation for patients presenting refractory hypoxemia or respiratory failure. Prone ventilation consists of providing mechanical ventilation with the patient in prone position. This is an additional therapy to treat severe hypoxemia caused by ARDS^(^ 8 ^)^.

The Prone Positioning in Severe Acute Respiratory Distress Syndrome (PROSEVA)^(^ 9 ^)^ trial provided scientific evidence of the effectiveness of the PP in the treatment of ARDS. The results of the randomized clinical trial conducted with 466 participants indicate that its early adoption (between 12 and 24 hours after the diagnosis of ARDS) for prolonged periods significantly decreased mortality in the intervention group. The rate of mortality in 28 days was 16% in the prone group and 32.8% in the control group (p<0.001), and 23.6% and 41.0%, respectively, in a 90-day period (p<0.001)^(^ 9 ^)^.

Considering that ARDS is the most severe complication of COVID-19, accounting for considerably high mortality rates, this study’s objective is to describe scientific evidence of the use of PP in the care provided to patients with acute respiratory failure caused by COVID-19.

## Method

This is a scoping review, which is characterized by the objective of mapping the main concepts of a field of knowledge, in this case, the Nursing field, and examining the extent, scope, and nature, in addition to summarizing and disseminating the results of studies, and identifying existing research gaps^(^ 10 ^)^.

The recommendations of the Joanna Briggs Institute Reviewer’s Manual^(^ 11 ^)^ were adopted. Additionally, the instrument titled PRISMA Extension for Scoping Reviews (PRISMA-ScR) was used in the elaboration of this study. This instrument is divided into seven domains and 22 items, providing recommendations regarding the title, abstract, introduction, method, results, discussion, conclusion, and financial support.

The search was conducted in the following databases: PubMed/MEDLINE, PMC, Science Direct, Web of Science, SCOPUS, Scientific Electronic Library Online (SciELO), and Google Scholar, between April and May 2020.

Scientific studies and other relevant articles available in the gray literature addressing the use of PP in the care provided to patients with acute respiratory failure caused by COVID-19 were considered.

The full texts of studies available free of charge and which answered the study question were included. Primary studies, systematic reviews, meta-analyses, guidelines, descriptive reports, official communications of governmental institutions, and studies addressing adult individuals were included without any language restrictions.

Studies that did not answer the study question or the objective of which was not PP related to respiratory failure caused by COVID-19 were excluded. Only studies published from December 2019 onwards were considered. The reason this timeframe was chosen is that this is when this pathology emerged and was identified.

The search process took place at three different points in time. First, a search was conducted in the Latin American and Caribbean Literature in Health Sciences (LILACS), Open Science Framework, and Cumulative Index to Nursing and Allied Health Literature (CINAHL) to identify titles and studies similar to that proposed here. No studies answering the study question were found in any of the databases. After identifying the originality of the topic and the need to produce evidence, we proceeded to the data collection.

Two researchers with a Master’s degree conducted an independent and blinded search. The starting time was determined and the search ended only when the possibilities of crossing were exhausted. Finally, the gray literature was consulted at a third point in time to identify manuals, consensus, and guidelines that potentially answered the research question. The question was established using the PCC strategy, according to the following.

P (Population) – Patients infected with COVID-19;

C (Concept) – Prone positioning;

C (Context) – Hospital care.

Hence, the following question was established: “What is the evidence available concerning the use of prone positioning in the care provided to patients with acute respiratory failure caused by COVID-19?”.

The following descriptors indexed in Medical Subject Headings (MeSH) were used in the search: 1. COVID-19; 2. new *coronavirus*; 3. 2019 nCOV; 4. SARS-CoV-2; 5. *Severe acute respiratory syndrome coronavirus* 2; 6. *Prone position*. The terms that would permit a broad search strategy regarding the subject, were used, namely: (“COVID-19” *OR* “*new coronavirus*” *OR* “2019 nCoV” *OR* “SARS-CoV-2” *OR* “*severe acute respiratory syndrome coronavirus* 2”) *AND* “*prone position*”.

After determining the descriptors and establishing the aforementioned strategy, the search in the databases/repositories was initiated. The databases were accessed through the CAPES periodicals portal, using the CAFe platform, a service that facilitates digital access through the use of a login registered at the university. An external search was also conducted in the gray literature, as recommended by the Reviewer’s Manual^(^ 11 ^)^.

After defining the sample, a protocol was adapted from the Cochrane Data collection form to extract data. The form addressed the following: country, year of publication, study objective, study design, eligibility criteria, institution where the intervention was conducted, population, methods used to implement the intervention, measures adopted to assess the intervention, outcomes, and complications accruing from the intervention.

The following information was extracted from the selected studies to answer the study question: 1) institution where the prone intervention was adopted; 2) criteria to adopt the PP; 3) PP duration; 4) main outcome and secondary outcome, and; 5) complications.

Note that the studies were classified in terms of levels of evidence, based on the Joanna Briggs Institute Levels of Evidence^(^ 11 ^)^: Level 1 – Experimental designs: 1.a) Systematic review of Randomized Controlled Trials; 1.b) Systematic review of randomized controlled trials, and other study designs; 1.c) Randomized controlled trials; 1.d) Pseudo-randomized controlled trials; Level 2 – Quasi-experimental designs: 2.a) Systematic review of quasi-experimental studies; 2.b) Systematic review of quasi-experimental and other lower study designs; 2.c) Quasi-experimental prospectively controlled study; 2.d) Pre-test and post-test or historic/retrospective control group study; Level 3 – Observational – Analytic Designs: 3.a) Systematic review of comparable cohort studies; 3.b) Systematic review of comparable cohort and other lower study designs; 3.c) Cohort study with control group; 3.d) Case – controlled study; 3.e) Observational study without a control group; Level 4–Observational–Descriptive Studies: 4.a) Systematic review of descriptive studies; 4.b) Cross-sectional study; 4.c) Case series Level; 4.d) Case study; Level 5 – Expert Opinion and Bench Research: 5.a) Systematic review of expert opinion; 5.b) Expert consensus; 5.c) Bench research/single expert opinion.

A descriptive analysis of data was performed, using relative and absolute frequencies. Data were characterized and results are presented in tables, figures, and graphs. Because this study does not involve human subjects, it did not require submission and approval by an Institutional Review Board. Additionally, there is minimum risk involved, as it is not experimental. Law No. 9,610/98 was fully complied with, intending to preserve and respect the ideas, concepts, and definitions adopted by the authors of the primary studies included in this review.

## Results

**Figure 1:**
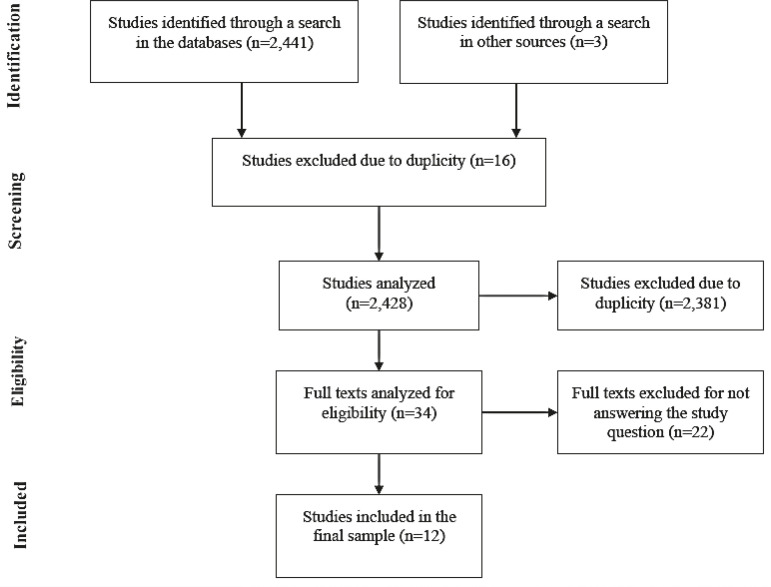
Flowchart of the study selection in this scoping review

Twelve of the 2,441 studies assessed were included in the final sample, as presented in Figure 1.

The sample is characterized by studies in the medical field (92%), predominantly conducted in the United States (33%), and published in 2020 (100%). As for the method adopted, reviews (42%) and expert consensus (42%) predominated.

Figure 2 presents the characterization of the studies included in the final sample according to the country of origin, study design, objective, main conclusions, and level of evidence according to the Joanna Briggs Institute.

**Figure 2:** Characterization of the studies composing the study sample

ID*	Country	Method	Objective	Main conclusions	LE^†^
A1^(^ 12 ^)^	USA	Case study	To verify the effect of prone positioning in one patient who tested positive for SARS-CoV-2.	PP is a vital part of the management plan and should be adopted early on to decrease mortality.	4d
A2^(^ 13 ^)^	China	Expert consensus	Establish guidelines to work with patients infected with 2019-nCoV.	If a patient who tested positive for COVID-19 develops ARDS, the adoption of invasive mechanical ventilation combined with prone positioning is necessary.	5b
A3^(^ 14 ^)^	Canada	Expert consensus	Serve as the basis to optimize supportive care for patients with COVID-19.	The implementation of PP is strongly recommended for adult patients with COVID-19, though it requires sufficient human resources and appropriate knowledge.	5b
A4^(^ 15 ^)^	Saudi Arabia	Narrative review	Describe the management of patients with ARDS caused by COVID.	Prone positioning, implemented among patients with severe ARDS, was associated with improved oxygenation, which was sustained after returning to the supine position.	n/e^‡^
A5^(^ 16 ^)^	Italy	Prospective cohort	Report the experience of a hospital with patients with COVID-19.	PP is suggested as an early treatment for COVID-19.	3c
A6^(^ 17 ^)^	Brazil	Expert consensus	PP in the treatment of acute respiratory distress in COVID-19.	Even though PP is a resource that improves oxygenation, caution is recommended when prescribing this positioning during the COVID-19 pandemic.	5b
A7^(^ 18 ^)^	United Kingdom	Expert consensus	Develop a flowchart to identify the benefits of prone positioning among patients with COVID-19.	Given the potential of PP to improve the oxygenation of patients with COVID-19, its use is recommended for all suitable and conscious patients in the ward.	5b
A8^(^ 19 ^)^	USA	Literature review	Describe the role of PP among patients with COVID-19.	The prone positioning can contribute to decrease mortality if implemented in the first hours after the manifestation of the disease.	n/e^‡^
A9^(^ 20 ^)^	Spain	Expert consensus	Share information regarding the treatment of patients infected with COVID-19.	The prone position improves the ventilation/perfusion ratio and prognosis. Some complications should be prevented though.	5b
A10^(^ 21 ^)^	USA	Literature review	Describe the clinical management of respiratory complications caused by COVID-19.	PP improves the ventilation/perfusion ratio, contributing to decrease mortality rates caused by the novel Coronavirus.	n/e^‡^
A11^(^ 22 ^)^	USA	Literature review	Disseminate a protocol regarding the clinical management of patients with sepsis caused by SARS-CoV-2.	The use of a POP is recommended to place patients in the prone position in all institutions. One should be attentive to absolute counter-indications.	n/e^‡^
A12^(^ 23 ^)^	Costa Rica	Scoping Review	Establish a guide for Nursing care provided to patients with COVID-19 in the prone position.	The prone positioning is an efficient alternative in the treatment of individuals with ARDS caused by COVID-19. Therefore, professional management is essential to provide quality nursing care to decrease complications and adverse events.	n/e^‡^

* ID = Identification;

† LE = Level of evidence;

‡ n/e = no evidence

The results show that 83% of the studies used PP among patients affected with severe acute respiratory failure caused by COVID-19 in Intensive Care Units, while the remaining studies proposed its adoption among clinically stable patients hospitalized in clinical wards.

The PAO_2_/FO_2_ ratio, oxygen saturation, and respiratory rate were the criteria most studies (92%) adopt to support decision-making concerning the implementation of PP. Gasometry parameters (FiO_2_, pH, pCO_2_, pO_2_ e HCO_3_) were also adopted (17%).

**Figure 3:**
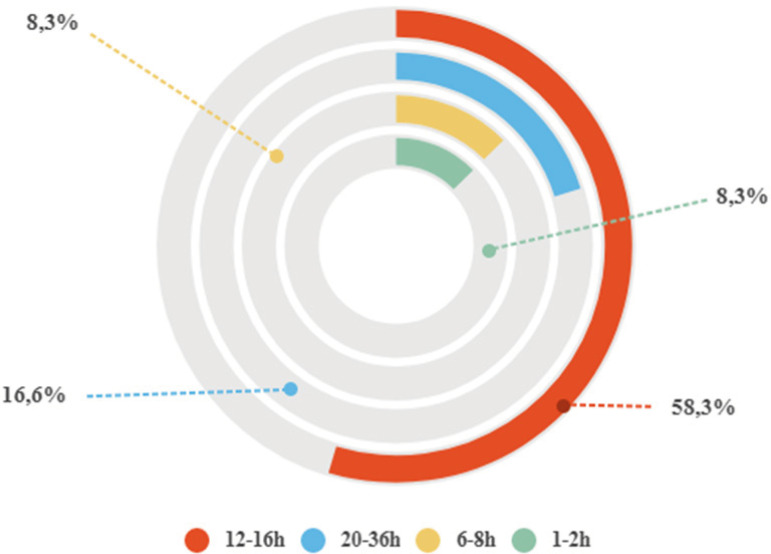
Duration of PP among patients with severe acute respiratory failure caused by COVID-19

There was disagreement regarding the duration of PP, though most studies (58.3%) suggest a period from 12 to 16 hours (Figure 3).

**Figure 4:**
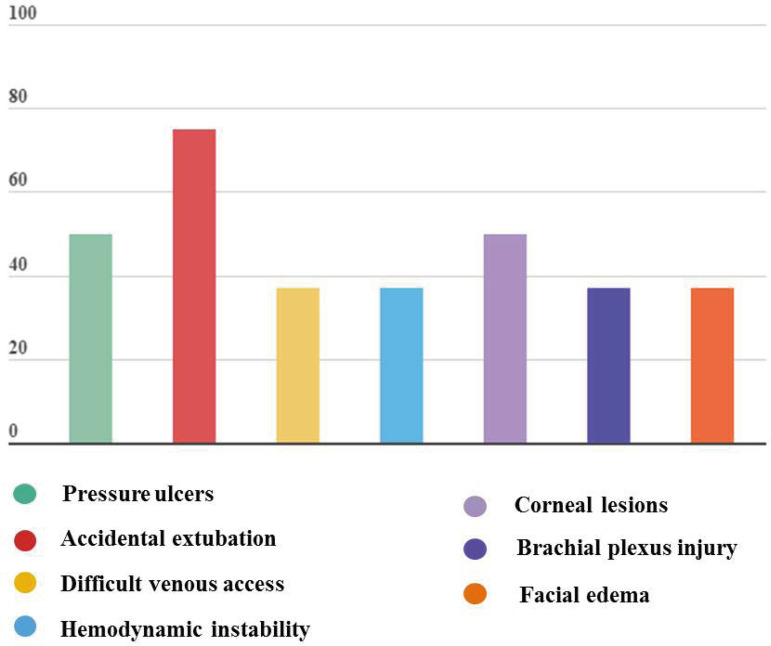
Complications accruing from the use of PP among patients with severe acute respiratory failure caused by COVID-19

Of the studies composing the sample, 67% report complications in the use of PP, the most frequent of which were: accidental extubation (78%), pressure ulcers (50%), and facial edema (50%) (Figure 4).

In addition to the aforementioned complications, there are other less frequent results, such as esophageal reflux, increased risk for pneumothorax and respiratory distress; increased risk for hemoptysis; tendency to hypersalivation and bruises in the perioral region.

**Figure 5:**
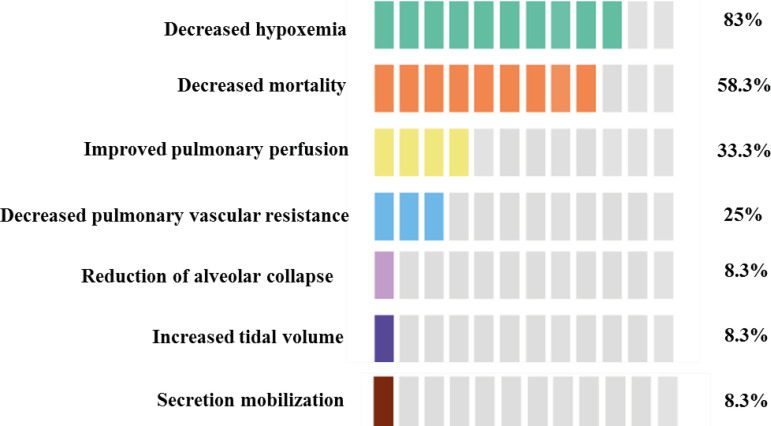
Main outcomes from the use of PP among patients with severe acute respiratory failure caused by COVID-19

Figure 5 presents the main primary and secondary outcomes identified in the studies composing the sample. Decreased hypoxemia, decreased mortality and improved pulmonary artery perfusion were the main outcomes from the adoption of PP in the studies.

Studies report that the early implementation of PP, especially among patients receiving mechanical ventilation, is an efficient strategy to reverse severe hypoxemia, resulting in decreased mortality. Its prescription, however, should be precisely assessed and potential complications weighted.

## Discussion

The sample adopted in this study was mostly composed of studies developed in the United States of America (A1, A8, A10, A11), reviews (A4, A8, A10, A11, A12) and expert consensus (A2, A3, A6, A7, A9). The predominance of the United States is likely explained by its current status as the epicenter of the pandemic, with 4,932,510 cases up to August 7^th^, 2020^(^ 2 ^)^. Additionally, the USA ranks first in the number of scientific papers published from 2013 to 2018, which characterizes it as the world scientific hub^(^ 24 ^)^.

As for the studies’ level of evidence in relation to the methods adopted, reviews provide robust evidence regarding a given topic. Additionally, they are original studies that do not require approval by an Institutional Review Board, which speeds up the process of writing and publishing papers^(^ 25 ^)^. In turn, when there is a lack of experimental studies or even reviews, consensuses written by renowned experts or experts with confirmed experience can be used to support and provide evidence-based practice^(^ 26 ^)^.

The use of PP among patients hospitalized in ICUs is explained by the severity of their conditions (A1, A2, A3, A4, A5, A6, A8, A9, A10, A11, A12), with a low PaO_2_/FiO_2_ ratio, revealing respiratory distress and negatively affecting noble organs such as brain, heart, and kidneys. ARDS of viral etiology stands out due to its high mortality, equal to about 50% of the cases, and is characterized by pulmonary edema of cardiogenic origin, causing hypoxemia and the need for invasive ventilatory support^(^ 27 ^)^.

Regarding the duration of prone positioning, recommendations vary but most studies recommend a minimum from 12 to 16 continuous hours (A2, A3, A4, A8, A9, A10, A11). The American Association of Critical-Care Nurses^(^ 28 ^)^ and the *Associação de Medicina Intensiva Brasileira* ^(^ 29 ^)^ (Brazilian Association of Intensive Care Medicine) recommend 16 hours of prone positioning for patients with ARDS receiving mechanical ventilation, a recommendation that is in line with this review’s findings.

Regarding complications, even though prone positioning was found to decrease pressure on bony prominences commonly injured in the supine or lateral positions^(^ 30 ^)^, PP exerts pressure on the frontalis and orbicularis muscles, chin, humerus, thorax, pelvis, and knees, causing several related adverse events^(^ 31 ^)^. Additionally, such pressure causes a heterogeneous distribution of blood and lymph flow in the face, as well as tissue ischemia and consequent necrosis, which results in the undesirable outcomes “pressure ulcers” and “facial edema”, identified in five of the studies in the sample (A2, A8, A9, A11, A12).

Direct pressure on the orbits, together with vascular changes, cause extraocular muscle impact, with the potential to culminate in conjunctival edema, bleeding, and even corneal injury, one of the complications that stands out in the sample (A9, A11, A12). A clinical trial reports that after ten minutes in the prone position, patients presented high intraocular pressure, as well as a greater risk of corneal ulceration. This type of damage may compromise eye function and require lifelong eye care, even though evidence shows that corneal abrasion and scleral wounds caused by prone positioning are generally self-limiting^(^ 32 ^)^.

Additionally, PP may cause traction on the humerus, either on its flexion or extension, leading to increased intraneural venous pressure, local edema, and impairment in the axoplasmic transmission of the elements that compose the brachial plexus^(^ 33 ^)^.

A case study of a patient placed in the prone position for a surgical procedure verified that, after five hours in this position, he developed brachial plexopathy. Studies suggest adopting measures such as cushions to reduce pressure on the pectoral muscles and prevent them from being pushed into the axillary fossa, pressing the plexus, as well as palpating the tendon of the pectoralis major muscle to monitor its tension^(^ 34 ^-^ 35 ^)^.

Among complications, six studies report the occurrence of accidental extubation (A2, A6, A7, A9, A11, A12). This complication is facilitated due to the spatial configuration of the position in relation to the airways, which makes them dilate due to the gravitational action on the local anatomical structures. Therefore, patients in the prone position may present an increased risk of displacing and twisting the orotracheal tube (OTT), leading to extubation^(^ 36 ^)^.

Another study, the objective of which was to report experiences in a COVID-19 ICU, also reports accidental extubation as one of the potential complications in prone positioning used to treat ARDS^(^ 37 ^)^. Therefore, studies^(^ 38 ^-^ 39 ^)^ recommend constant and vigilant monitoring of OTT and timely action when this problem occurs, as it may aggravate an already critical condition, imposing even greater risks on patients^(^ 38 ^)^.

Likewise, PP presents some peculiar hemodynamic challenges. One observational prospective study verified that compression of the abdomen during PP may restrict blood flow from the inferior vena cava, causing venous engorgement and consequent decrease in the cardiac output^(^ 40 ^)^. In the context of a patient with severe acute respiratory failure caused by COVID-19, this may be a desired outcome to achieve decreased myocardial work and prevent cardiac factors associated with respiratory failure. The combination of arterial hypotension, increased intra-abdominal pressure, and hypovolemia in patients in the prone position may lead to poor perfusion to multiple systems and cause hemodynamic instability though, a complication reported in three studies in the sample (A8, A11, A12)^(^ 41 ^)^.

Regarding the pathophysiological mechanism of COVID-19, in addition to acute respiratory distress syndrome, there are also acute kidney complications and multiple organ failure^(^ 37 ^,^ 42 ^)^. Therefore, many of these patients require extensive support encompassing varied procedures that require venous access. Nonetheless, as previously mentioned, pronation adopted in this circumstance to manage ARDS complicates obtaining venous access, as shown by studies A8, A9, and A11.

This finding is corroborated by a case report that used popliteal venous access to enable renal replacement therapy in a critical patient with COVID-19 who was in PP. This procedure was justified by the difficulty in finding another site to puncture the intravenous route^(^ 43 ^)^.

Therefore, the literature shows that PP improves gas exchange in approximately two-thirds of patients with ARDS because it works as a recruitment maneuver with long-term effects, which leads to improved oxygenation. This maneuver explores gravity and repositioning of the heart in the chest to recruit pulmonary perfusion ratio and arterial oxygenation^(^ 44 ^)^.

The prone position enables a redistribution of alveolar ventilation and perfusion. With a decrease in the effects of compression that favor atelectasis, pleural pressure is reduced, as well as transpulmonary pressures, and thus, alveolar recruitment can be achieved in atelectatic areas^(^ 44 ^)^. These PP mechanisms clarify the outcomes reported by the studies in the sample.

It is known that pulmonary involvement caused by the SARS-CoV-2 infection is uniform, causing an increase in lung volume, as a result of the edema, a result of the inflammatory process. Considering gravitational basilar reasoning, researchers^(^ 45 ^)^ used tomographic resources and verified that the dorsal part of the lungs suffers greater impact when in the supine position, caused by increased lung volume, which generates collapse among dependent regions.

A case study^(^ 46 ^)^ conducted with a patient infected with SARS-CoV-2 compared the tomography performed in the patient in the supine position *versus* prone position. The supine positioning revealed a significant increase in the extension and accentuation of opacities, with pulmonary consolidation and atelectasis of the right lower lobe. The tomography performed in the prone position showed partial recovery of the pulmonary parenchyma and a decrease in the previous pulmonary consolidations^(^ 46 ^)^.

Pronation is a strategy that tends to decrease the impact caused by increased lung weight, caused by the edema, under important regions, enabling improved oxygenation^(^ 47 ^)^, as discussed in studies A1, A3, A4, A5, A6, A7, A9, A10, A11, and A12. Additionally, studies^(^ 48 ^-^ 49 ^)^ show an increase in tidal volume to be responsible for improved oxygenation in PP, which is in line with study A8.

Improved oxygenation is the effect most frequently expected and discussed in studies when PP is adopted. In addition to what we discussed previously, this effect also takes place due to a decrease in the various factors that contribute to alveolar collapse, such as redistribution of alveolar ventilation, reordering of perfusion, and reducing dorsal lung compression^(^ 47 ^,^ 50 ^)^. Note that improved pulmonary perfusion is an outcome reported in studies A1, A4, A5, and A10.

The case study^(^ 12 ^)^ of a patient with severe acute respiratory failure caused by COVID-19 reports that, after 12 hours in the prone position, the patient progressed from the initial environmental O_2_ saturation of 85% to 95% at rest and 90% when walking. Clinical trials report that oxygenation is improved in the prone group when compared to a group of patients in the supine position, with an increased PaO_2_/FiO_2_ ratio^(^ 9 ^,^ 51 ^)^.

In contrast, a study^(^ 52 ^)^ that analyzed the oxygenation of ten critical patients who tested positive for SARS-CoV-2 and were intubated and receiving mechanical ventilation reports improved PaO_2_/FiO_2_ ratios in the supine position when compared to the long test of patients in the prone position (*p*=0.034).

Another important aspect is the role that the position of the rib cage plays in transpulmonary pressure. In PP, the rib cage presents a rectangular format, which results in decreased alveolar collapse, according to study A8.

Additionally, it is known that the cardiac muscle plays an important role in the lungs when in normal physiological conditions. This effect in a patient with respiratory failure due to COVID-19 may be even more important due to an increase in the right cardiac chamber, secondary to pulmonary hypertension, and hypoxic vasoconstriction, which results in increased pulmonary vascular resistance (A2, A7, and A8).

Likewise, two studies show that PP promotes the mobilization of secretions, which may improve the oxygenation of patients (A1 and A2). The reason is that PP enables improved drainage of secretion from the airways, which further promotes a reduction in the risk of respiratory infection associated with mechanical ventilation.

One study^(^ 53 ^)^ reports that PP greatly impacts the cardiopulmonary physiology, being a useful maneuver accessible for most ICUs.

PP improves pulmonary mechanics and gas exchange and guidelines currently recommended it^(^ 54 ^-^ 55 ^)^. Therefore, PP should be considered in the initial stages of respiratory failure, considering that evidence suggests that the early implementation of prolonged ventilation in PP decreases mortality among patients with severe ARDS caused by COVID-19 (A1, A6, A7, A9, A10, and A11).

Thus, PP can improve oxygenation and compliance of the pulmonary system of patients with severe acute respiratory syndrome caused by COVID-19^(^ 56 ^)^. It can also decrease mortality rates in the subgroup with severe ARDS and causes few complications when its positive outcomes are considered^(^ 57 ^)^.

This study presents limitations such as a lack of studies in the sample with high levels of evidence, such as randomized clinical trials. This gap, however, is explained by the fact this is a recent disease, with little time for studies that require long follow-up periods.

This study’s social contribution refers to an analysis of the most recent studies adopting PP among patients with respiratory failure caused by COVID-19, a disease of global repercussions, the impact of which in the health and economy spheres has caused profound changes in society. The results found here support the improvement of work processes in health and nursing, for consequent improvement of the care provided to the population.

As for its scientific relevance, this study contributes with an attempt to deepen the subject, aiming to fill this gap in the literature addressing the topic. Additionally, scoping reviews are useful to examine emerging evidence regarding topics that still lack robust evidence, as is the case of the novel Coronavirus.

## Conclusion

This study’s objective was to describe scientific evidence concerning the use of PP in the care provided to patients with acute respiratory failure caused by COVID-19. The sample was composed of 12 studies, which showed the use of PP, mainly in ICUs, with a duration from 12 to 16 hours.

The criteria used by the health staff to implement PP include the PAO_2_/FiO_2_ ratio, oxygen saturation, and respiratory rate. Complications caused by PP were also identified: accidental extubation, pressure ulcers, and facial edema were the most frequently reported.

The positive outcomes outweighed the complication though. Thus, considering the evident decrease in hypoxemia and mortality rates, its use is recommended for patients with respiratory failure caused by SARS-CoV-2.

That said, sustained improvement in oxygenation requires various cycles of pronation, a factor that may potentially overload the work of the health staff. Indeed, the studies suggest that having an appropriate number of trained workers and specific institutional protocols to ensure patient safety in this context is required.
